# Perinatal Outcomes by Mode of Assisted Conception and Sub-Fertility in an Australian Data Linkage Cohort

**DOI:** 10.1371/journal.pone.0080398

**Published:** 2014-01-08

**Authors:** Jennifer L. Marino, Vivienne M. Moore, Kristyn J. Willson, Alice Rumbold, Melissa J. Whitrow, Lynne C. Giles, Michael J. Davies

**Affiliations:** 1 Department of Obstetrics and Gynaecology, The University of Melbourne, Royal Women's Hospital, Parkville, Victoria, Australia; 2 Discipline of Public Health, The University of Adelaide, Adelaide, South Australia, Australia; 3 Robinson Institute, The University of Adelaide, Adelaide, South Australia, Australia; 4 Discipline of Obstetrics and Gynaecology, The University of Adelaide, Adelaide, South Australia, Australia; VU University Medical Center, The Netherlands

## Abstract

**Background:**

Fertility treatment is associated with increased risk of major birth defects, which varies between in vitro fertilisation (IVF) and intracytoplasmic sperm injection (ICSI), and is significantly reduced by embryo freezing. We therefore examined a range of additional perinatal outcomes for these exposures.

**Methods:**

All patients in South Australia receiving assisted conception between Jan 1986–Dec 2002 were linked to the state-wide perinatal collection (all births/stillbirths ≥20 weeks gestation or 400 g birth weight, n = 306 995). We examined stillbirth, mean birth weight, low birth weight (<2500 g, <1500 g), small size for gestational age (<10th percentile, <3rd percentile), large size for gestational age (>90th percentile), preterm birth (32–<37 weeks, <32 weeks gestation), postterm birth (≥41 weeks gestation), Apgar <7 at 5 minutes and neonatal death.

**Results:**

Relative to spontaneous conceptions, singletons from assisted conception were more likely to be stillborn (OR = 1.82, 95% Confidence Interval (CI) 1.34–2.48), while survivors as a group were comprehensively disadvantaged at birth, including lower birth weight (−109 g, CI −129–−89), very low birth weight (OR = 2.74, CI 2.19–3.43), very preterm birth (OR = 2.30, CI 1.82–2.90) and neonatal death (OR = 2.04, CI 1.27–3.26). Outcomes varied by type of assisted conception. Very low and low birth weight, very preterm and preterm birth, and neonatal death were markedly more common in singleton births from IVF and to a lesser degree, in births from ICSI. Using frozen-embryos eliminated all significant adverse outcomes associated with ICSI but not with IVF. However, frozen-embryo cycles were also associated with increased risk of macrosomia for IVF and ICSI singletons (OR = 1.36, CI 1.02–1.82; OR = 1.55, CI 1.05–2.28). Infertility status without treatment was also associated with adverse outcomes.

**Conclusions:**

Births after assisted conception show an extensive range of compromised outcomes that vary by treatment modality, that are substantially reduced after embryo freezing, but which co-occur with an increased risk of macrosomia.

## Introduction

While birth outcomes after assisted conception have been studied, particularly descriptively, and subject to meta-analysis [1]–[5], few studies have been designed or scaled to permit comparisons of outcomes among different types of assistance [6], and separately for singletons and twins. Additionally, although fertility impairment itself has a negative impact on birth outcomes [7]–[10], only two studies [11], [12], both restricted to singletons, have examined the effects of subfertility and mode of conception in the same patient population.

We have recently demonstrated that treatment modality, subfertility and embryo freezing each contribute to the risk of major birth defects [13]. Here we examine the impact of assisted conception method and fertility impairment on a range of perinatal outcomes other than major birth defects, separately for singleton and twin births, and with adjustment for common confounders.

## Methods and Materials

### Data source

As previously described [13], we created a database linking all infertility treatment in South Australia between January 1986 and December 2002 to the contemporaneous state perinatal collection. The perinatal collection details all live births and stillbirths of at least 20 weeks' gestation or 400 g birth weight (around 20,000/year), including birth weight, gestational age, and maternal pre-existing and gestational medical conditions. The two clinics in the state licensed to manipulate gametes (the University of Adelaide and Flinders University) provided data for all patient visits for the period. The resulting de-identified database includes information for 327,378 registered births (321,210 spontaneously conceived).

### Exclusions

There were 20,383 exclusions. The majority (n = 18,420, 90.4%) were pregnancies among mothers under 20 years of age as only 2 of these pregnancies were conceived with infertility treatment. Births of higher-order multiplicity (triplet and quadruplet) were also excluded because appropriate size references do not exist. As all analyses were adjusted for infant sex of the baby, 311 births and terminations of indeterminate sex and where the sex was unknown were also excluded (n = 304 (0.1%) conceived spontaneously and n = 7 (0.1%) conceived with infertility treatment).

### Exposures and covariates

Births after any assisted conception (n = 5,949) were compared to those after spontaneous conception (n = 301,046). The spontaneous conception group was further classified into births to women with no history of infertility in their records and no infertility treatment (“spontaneous conception, fertile”, n = 298,952), births to women who had a recorded diagnosis of infertility but no associated assisted conception treatment from a specialist clinic (“spontaneous conception, IF DX”, n = 767), and births as a result of spontaneous conception in women with a previous birth from assisted conception (“spontaneous conception, IF TX”, n = 1,327).

Types of assisted conception considered were: donor oocyte procedures, gamete intrafallopian transfer (GIFT), intrauterine insemination (IUI), in vitro fertilization (IVF) with fresh-embryo cycles, IVF with frozen-embryo cycles, intracytoplasmic sperm injection (ICSI) with fresh-embryo cycles, ICSI with frozen-embryo cycles, minimal medical intervention and ovulation induction (OI) only. Conceptions with donor oocytes were grouped together irrespective of mode of assisted conception. Conceptions resulting from IUI only (n = 702) and IUI after cancellation of IVF (n = 11) or ICSI (n = 2) were grouped.

The IVF with fresh-embryo transfer group (n = 1519) included pregnancies arising from zygote intrafallopian transfer (ZIFT) or tubal embryo transfer (TET) (n = 9) (as such treatments have medication and culture regimens similar to those of IVF), and all natural-cycle (n = 12) and OI (n = 1,498) IVF pregnancies not specifying embryo transfer. All pregnancies involving donor embryos (n = 9), imported embryos (n = 10), and IVF specifying “embryo transfer” with or without mentioning freezing (respectively n = 39, n = 546) were included in the IVF with frozen-embryo transfer group (n = 604).

The minimal medical intervention group (n = 710) included conceptions in women who were tracking their ovulatory cycles in anticipation of starting treatment or who were being monitored for other reasons (n = 167), to women who received donor insemination (n = 466), to a woman whose primary treatment was weight loss, to a woman whose husband was treated with human chorionic gonadotrophin (hCG); conservatively, these women were counted among this group because it is possible that some received hormonal medication to adjust ovulation timing, even though this was not recorded in their medical records (for example, medication prescribed outside of the licensed fertility clinic). As well, conceptions during ovarian suppression regimens (n = 69) and after tubal or ovarian surgery (n = 6) were counted in this group.

The OI only group (n = 406) included pregnancies among those who received only an OI regimen (clomiphene citrate +/− bromocryptine) (n = 386), those who were tracking their cycles and receiving OI regimens (n = 7), and those whose IUIs were cancelled for ovarian hyperstimulation (n = 4). The group also included pregnancies arising during IUI control cycles (n = 6), and from donor insemination after IVF cancellation (n = 3), as it was likely that such women had begun OI or that ovarian hyperstimulation was the reason for cancellation.

### Outcomes

Birth outcomes considered were stillbirth, birth weight, very low birth weight (VLBW, <1,500 g), low birth weight (LBW, <2,500 g), very preterm birth (VPTB, <32 weeks gestation), preterm birth (PTB, <37 weeks gestation), postterm birth (>41 weeks gestation), very small size for gestational age (VSGA, <3rd percentile), small size for gestational age (SGA, <10th percentile), large size for gestational age (LGA, >90th percentile), Apgar score <7 at 5 minutes, and neonatal death. Analyses of birth outcomes other than stillbirth were restricted to liveborn singletons (n = 296,401) and twins (n = 8,824). Australian national standards for sex-specific birth weight centiles by gestational age were used to define VSGA, SGA, and LGA separately for singletons [14] and twins [15].

Analyses were stratified by plurality. Generalized estimating equations were used to produce odds ratios accounting for clustering of births within women. All analyses were adjusted for maternal age, parity, and infant sex; those for twin analyses were further adjusted for same-sex twinship to reduce the role of fetal zygosity. Within some assisted conception groups, certain outcomes were too rare for stratification. In these circumstances, groups were combined within type of conception first (i.e. singletons and twins were grouped together) and then if necessary, assisted conception groups were combined according to similarity of treatment. Where singletons and twins were grouped, models were fitted to force the estimates for modality to be the same for both groups, with multiplicity-specific comparators (i.e. the reference group for singletons born after the modality was spontaneously conceived singletons, and for twins born after the modality was spontaneously conceived twins.) All statistical analyses were performed using SAS 9.2 (©2009 SAS Institute Inc., Cary, NC).

### Ethics approval

Approval for the study was obtained from the ethics committees of the South Australian Department of Health, the University of Adelaide, and Flinders University. Individual level consent was not required.

## Results

Table 1 summarizes characteristics of live births and stillbirths by mode of conception. For both types of birth, women who used assisted conception were significantly older and less likely to have had a previous birth than women who conceived spontaneously.

**Table 1:** Characteristics of live births and stillbirths by mode of conception.

	Live births, spontaneous	Live births, assisted conception	Stillbirths, spontaneous	Stillbirths, assisted conception
N	299356	5869	1690	80
Maternal age, years, mean (SD)† ‡	29.2 (4.8)	33.0 (4.2)	29.4 (5.3)	33.2 (4.2)
Age, N (%)† ‡				
20–24 years	62307 (20.8) 112974 (37.7)	132 (2.2)	388 (23.0)	1 (1.3)
25–29 years		1306 (22.3) ()	579 (34.3)	16 (20.0)
30–34 years	87985 (29.4)	2586 (44.1)	440 (26.0)	37 (46.3)
35–39 years	31167 (10.4)	1541 (26.3)	228 (13.5)	22 (27.5)
40+	4923 (1.6)	304 (5.2)	55 (3.3)	4 (5.0)
Primigravid, N (%)†	202218 (67.6)	3658 (62.3)	977 (57.8)	48 (60.0)
Parity, N (%)† ‡				
0	112216 (37.5)	3824 (65.2)	712 (42.1)	63 (78.8)
1	108474 (36.2)	1604 (27.3)	471 (27.9)	9 (11.3)
2+	78666 (26.3)	441 (7.5)	507 (30.0)	8 (10.0)
Sex ratio (M∶F)	1.061	1.013	1.069	0.951
Multiple gestation, N (%)† ‡	7227 (2.4)	1808 (30.8)	150 (8.9)	42 (52.5)
Multiple birth, N (%)† ‡	7215 (2.4)	1609 (27.4)	147 (8.7)	35 (43.8)

SD: standard deviation. IQR: interquartile range.

Significantly different between groups for the comparison between spontaneous and assisted conception live births (p<0.05).

Significantly different between groups for the comparison between spontaneous and assisted conception stillbirths (p<0.05).

### Stillbirth

Table 2 shows associations between assisted conception and stillbirth. Singletons from any assisted conception treatment were almost twice as likely to be stillborn than those conceived spontaneously.

**Table 2:** Stillbirth among singletons and twins by mode of conception.

	Singletons				Twins			
	All births	Stillborn		(OR 95% CI)	All births	Stillborn		OR (95% CI)
	N	N	%		N	N	%	
Spontaneous	293684	1543	0.5	Ref	7362	147	2.0	Ref
Any assisted conception	4305	45	1.1	**1.82 (1.34,2.48)**	1644	35	2.1	1.06 (0.65,1.72)
Spontaneous conception, fertile	291793	1524	0.5	Ref	7159	143	2.0	Ref
Spontaneous conception, IF DX*	597	14	2.4	**4.11** # **(2.33,7.27)**	170	4	2.4	1.09# (0.36,3.29)
Spontaneous conception, IF TX**	1294	5	0.4	0.64∧ (0.24,1.68)	33	0	0.0	0.64∧ (0.24,1.68)
Donor oocyte	69	1	1.5	1.20∧ (0.14,10.4)	16	0	0.0	1.20∧ (0.14,10.4)
GIFT	317	5	1.6	**2.82 (1.15,6.93)**	219	7	3.2	1.67 (0.62,4.55)
IUI	575	4	0.7	1.21 (0.44,3.33)	140	2	1.4	0.65 (0.12,3.52)
IVF with fresh-embryo cycles	961	13	1.4	**2.35 (1.34,4.11)**	558	15	2.7	1.39 (0.69,2.79)
IVF with frozen-embryo cycles	454	6	1.3	2.31 (0.997,5.37)	150	3	2.0	0.97 (0.19,4.91)
ICSI with fresh-embryo cycles	703	10	1.4	**2.46 (1.29–4.68)**	386	6	1.6	0.84 (0.28,2.49)
ICSI with frozen-embryo cycles	220	2	0.9	0.74∧ (0.15,3.70)	65	0	0.0	0.74∧ (0.15,3.70)
Minimal medical intervention	664	3	0.5	0.75 (0.22,2.57)	46	1	2.2	1.41 (0.20,9.83)
OI only	342	1	0.3	0.52 (0.07,4.18)	64	1	1.6	0.72 (0.07,7.44)

Odds ratios account for clustering within mother, and are adjusted for maternal age, parity, baby's sex and whether twins same-sex.

IF DX: Births to women who had a recorded diagnosis of infertility but no assisted conception treatment from a specialist clinic.

IF TX: Births as a result of spontaneous conception in women with a previous birth from assisted conception.

Singletons and twins combined because of small numbers.

^#^ Significant difference between effect for singletons and twins.

Compared to spontaneous conceptions in women with no recorded infertility history or infertility treatment (spontaneous conception, fertile group), spontaneous conceptions to women with a recorded infertility diagnosis but no treatment (spontaneous conception, IF DX group), and conceptions achieved with GIFT, with IVF with fresh-embryo cycles, and with ICSI with fresh-embryo cycles were more likely to end in stillbirth. Conceptions resulting from IVF with frozen-embryo cycles were more likely to be stillborn than the fertile group, but the difference did not reach statistical significance. There were no differences in stillbirth risk among twins by mode of conception.

### Liveborn singletons

Table 3 shows associations between birth outcomes and mode of conception among singletons. Compared to births from spontaneous conceptions, births from any assisted conception were significantly lighter at birth, more often born VLBW, LBW, very preterm, preterm, VSGA, and SGA, and were more likely to die in the neonatal period. They were significantly less likely to be born postterm but did not differ in likelihood of LGA or an Apgar score <7 at 5 minutes.

**Table 3:** Perinatal outcomes among singleton live births by mode of conception.

	All	Birth weight*		VLBW*		LBW*		VPTB		PTB		Postterm birth		VSGA*		SGA*		LGA		5′ Apgar <7**				Neonatal death				
	N	Mean	Mean diff	N	OR	N	OR	N	OR	N	OR	N	OR	N	OR	N	OR	N	OR	N		OR		N		OR		
		SD	95%CI	%	95%CI	%	95%CI	%	95%CI	%	95%CI	%	95%CI	%	95%CI	%	95%CI	%	95%CI	%		95%CI		%		95%CI		
Spontaneous	292141	3399	Ref	2212	Ref	13667	Ref	2495	Ref	13572	Ref	39575	Ref	8917	Ref	29638	Ref	28969	Ref	4151		Ref		823		Ref		
		553		0.8		4.7		0.9		4.7		13.6		3.1		10.2		9.9		1.4				0.3				
Any assisted conception	4260	3259	**−109**	93	**2.74** #	400	**1.98** #	86	**2.30** #	337	**1.64** #	434	**0.65**	179	**1.30**	542	**1.22** #	357	0.91	72		1.17		21		**2.04** #		
		641	**−129,−89**	2.2	**2.19,3.43**	9.4	**1.77,2.20**	2.0	**1.82,2.90**	7.9	**1.46,1.84**	10.2	**0.59,0.72**	4.2	**1.12,1.52**	12.7	**1.11,1.33**	8.4	0.81,1.02	1.7		0.92,1.49		0.5		**1.27,3.26**		
Spontaneous conception, fertile	290269	3400	Ref	2160	Ref	13527	Ref	2448	Ref	13448	Ref	39369	Ref	8860	Ref	29465	Ref	28752	Ref	4119		Ref		806		Ref		
		552		0.7		4.7		0.8		4.6		13.6		3.1		10.2		9.9		1.4				0.3				
Spontaneous conception, IF DX***	583	3081	**−256**	39	**8.99** #	98	**3.67** #	34	**6.96** #	76	**2.76** #	78	0.88	36	**1.81**	90	**1.34**	41	0.78	19		**2.18**		10		**6.94**		
		811	**−318,−194**	6.7	**6.38,12.7**	16.8	**2.94,4.58**	5.8	**4.88,9.92**	13.0	**2.17,3.52**	13.4	0.70,1.12	6.2	**1.29,2.54**	15.4	**1.06,1.69**	7.0	0.57,1.07	3.3		**1.38,3.45**		1.7		**3.59,13.4**		
Spontaneous conception, IF TX****	1289	3456	2	13	1.49	42	0.83	13	1.30	48	0.87	128	**0.81**	21	**0.64** ∧	83	**0.77**	176	1.12	13		0.76∧		7		1.69∧		
		571	−29,33	1.0	0.87,2.54	3.3	0.62,1.11	1.0	0.77,2.21	3.7	0.65,1.17	9.9	**0.67,0.99**	1.6	**0.42,0.97**	6.4	**0.62,0.95**	13.7	0.95,1.33	1.0		0.42,1.38		0.5		0.76,3.74		
Donor oocyte	68	3189	−171	4	**8.31**	9	**2.77**	4	**7.25**	5	1.49	8	0.73	1	0.35∧	7	0.88	4	0.69	2		1.32∧		†		-		
		790	−369,27	5.9	**3.06,22.6**	13.2	**1.38,5.55**	5.9	**2.65,19.8**	7.4	0.61,3.67	11.8	0.32,1.67	1.5	0.05,2.45	10.3	0.40,1.93	5.9	0.26,1.84	2.9		0.31,5.67						
GIFT	312	3141	**−211**	12	**5.12** #	33	**2.23** #	10	**3.83** #	13	0.82	24	**0.47**	18	**1.84**	62	**2.12** #	21	0.76	8		1.79		1		1.42		
		663	**−288,−135**	3.9	**2.84,9.25**	10.6	**1.56,3.17**	3.2	**2.01,7.31**	4.2	0.46,1.47	7.7	**0.31,0.72**	5.8	**1.16,2.91**	19.9	**1.60,2.80**	6.7	0.49,1.18	2.6		0.88,3.66		0.3		0.17,11.6		
IUI	571	3283	**−89**	10	**2.31**	49	**1.81**	8	1.65	48	**1.76** #	67	**0.74**	34	**1.81**	62	0.96	43	0.83	11		1.34		0		0.28∧		
		609	**−137,−41**	1.8	**1.23,4.33**	8.6	**1.35,2.42**	1.4	0.82,3.31	8.4	**1.31,2.36**	11.7	**0.57,0.97**	6.0	**1.27,2.58**	10.9	0.74,1.26	7.5	0.60,1.14	1.9		0.74,2.44		0.0		0.04,1.99		
IVF with fresh-embryo cycles	948	3119	**−248** #	37	**4.96** #	133	**3.07** #	33	**3.98** #	99	**2.20** #	86	**0.55**	43	1.36	146	**1.50**	50	**0.56**	19		1.38		11		**4.92** #		
		697	**−293,−204**	3.9	**3.52,6.99**	14.0	**2.55,3.69**	3.5	**2.78,5.71**	10.4	**1.79,2.70**	9.1	**0.44,0.69**	4.5	0.99,1.86	15.4	**1.26,1.80**	5.3	**0.42,0.74**	2.0		0.7,2.19		1.1		**2.65,9.11**		
IVF with frozen-embryo cycles	448	3375	−3	7	2.00	33	**1.58** #	8	2.01	42	**2.02** #	33	**0.48**	10	0.75	28	**0.59**	58	**1.36**	10		1.59		3		0.77∧		
		647	−61,55	1.6	0.94,4.25	7.4	**1.12,2.23**	1.8	0.98,4.12	9.4	**1.49,2.75**	7.4	**0.34,0.68**	2.2	0.41,1.36	6.3	**0.41,0.85**	13.0	**1.02,1.82**	2.2		0.85,2.97		0.6		0.21,2.80		
ICSI with fresh-embryo cycles	693	3220	**−139**	8	1.54	66	**1.96** #	8	1.41	57	**1.63** #	85	**0.76**	33	**1.42** #	114	**1.54** #	44	**0.73**		6		0.59		4		2.54	
		589	**−183,−95**	1.2	0.78,3.04	9.5	**1.52,2.52**	1.2	0.71,2.77	8.2	**1.24,2.15**	12.3	**0.60,0.95**	4.8	**1.003,2.01**	16.5	**1.26,1.89**	6.4	**0.53,0.997**	0.9		0.26,1.32		0.6		0.91,7.06		
ICSI with frozen-embryo cycles	218	3459	**81**	0	0.77∧	8	0.78	0	1.00∧	11	1.08	22	0.67	4	0.49∧	16	0.71	30	**1.55**	1		0.33		1		1.90		
		491	**19,143**	0.0	0.24,2.45	3.7	0.40,1.52	0	0.37,2.70	5.1	0.60,1.94	10.1	0.43,1.05	1.8	0.19,1.27	7.3	0.44,1.14	13.8	**1.05,2.28**	0.5		0.04,2.46		0.5		0.23,15.9		
Minimal medical intervention	661	3362	−6	9	1.81	39	1.21	10	1.85	34	1.06	71	**0.71**	19	0.86∧	67	0.91	69	1.16	7		0.65∧		1‡		0.20∧		
		590	−52,39	1.4	0.92,3.58	5.9	0.87,1.68	1.5	0.98,3.49	5.1	0.74,1.51	10.7	**0.55,0.91**	2.9	0.54,1.36	10.1	0.69,1.19	10.4	0.90,1.50	1.1		0.30,1.38		0.1		0.02,1.85		
OI only	341	3329	−43	6	2.31	30	**1.79**	5	1.68	28	**1.72**	38	0.72	17	1.51	40	1.16	38	1.30	8		1.65						
		648	−109,24	1.8	0.99,5.39	8.8	**1.22,2.63**	1.5	0.65,4.33	8.2	**1.16,2.56**	11.1	0.52,1.004	5.0	0.91,2.50	11.7	0.85,1.59	11.1	0.93,1.83	2.4		0.83,3.29						

Odds ratios account for clustering within mother, and are adjusted for maternal age, parity, and baby's sex.

Bold print signifies p<0.05.

Twenty-four births were missing birth weight information.

414 births were missing 5 minute Apgar score information.

^#^ Significant difference between effect for singletons and twins

IF DX: Births to women who had a recorded diagnosis of infertility but no assisted conception treatment from a specialist clinic.

IF TX: Births as a result of spontaneous conception in women with a previous birth from assisted conception.

Data for neonatal death occurring in donor oocyte pregnancies collapsed in IVF and ICSI categories due to small numbers

Data for neonatal death occurring in the groups minimal medical intervention and ovulation induction only combined due to small numbers

Singletons and twins combined because of small numbers (comparator is singletons)

Low birth weight (LBW): <2500 g; Very low birth weight (VLBW): <1500 g; Preterm birth (PTB): 32–37 weeks; Very preterm (VPTB): <32 weeks; Postterm: >41 weeks;

SGA <10th percentile, Roberts [14]; VSGA <3rd percentile, Roberts [14]; LGA: >90th percentile, Roberts [14].

Relative to the spontaneous conception, fertile group, infants of women with a recorded infertility diagnosis but no treatment were significantly lighter, more likely to be born VLBW or LBW, very preterm or preterm, and VSGA, were more likely to have a an Apgar score <7 at 5 minutes, and were more likely to die in the neonatal period. Infants of women with a previous assisted conception birth (spontaneous conception, IF TX group) were less likely to be born postterm, VSGA or SGA than the spontaneous conception, fertile group. However there were no other differences in outcomes between these groups.

Compared to the spontaneous conception, fertile group, infants as a result of donor oocytes did not differ significantly in mean birth weight, but were at increased risk of being born VLBW, LBW or very preterm. Infants resulting from GIFT were lighter, and were more likely to be born VLBW, LBW, very preterm, VSGA, and SGA, and less likely to be postterm. IUI infants were lighter at birth and more likely to be VLBW, LBW, preterm, or VSGA, and less likely to be postterm.

Infants from IVF with fresh-embryo cycles were lighter at birth and more likely to be born VLBW, LBW, very preterm or preterm, SGA, and were more likely to die in the neonatal period. They were also less likely to be postterm or LGA than those in the spontaneous conception, fertile group. Among infants from IVF with frozen-embryo cycles, there was no increased risk of neonatal death, VLBW or VPTB, however the associations with LBW, PTB, and SGA remained, and there was an increased risk of LGA. Infants from ICSI with fresh-embryo cycles were lighter, had increased risks of LBW, VSGA, SGA and a decreased risk of being postterm or LGA. However, the associations between ICSI and adverse perinatal outcomes were no longer present for ICSI with frozen-embryo cycles. In fact, these infants were significantly heavier and more likely to be LGA than those in the spontaneous conception, fertile group.

Infants of women in the minimal medical intervention group were less likely to be postterm and did not otherwise differ from the spontaneous conception, fertile group infants. Use of ovulation induction alone was associated with an increased risk of LBW and PTB.

### Liveborn twins

Table 4 shows associations between outcomes by mode of conception among twins. Compared to those conceived spontaneously, twin babies conceived with any assisted conception were born lighter and were more often born VLBW or very preterm. Relative to twins born to the spontaneous conception, fertile group, those in the spontaneous conception, IF DX group were born lighter and were more likely to be VLBW and die in the neonatal period. Twins conceived spontaneously in women with a previous assisted conception birth were less likely to be VSGA than twins in the spontaneous conception, fertile group.

**Table 4:** Perinatal outcomes among twin live births by mode of conception.

	All	Birth weight		VLBW		LBW		VPTB		PTB		Postterm birth**		VSGA		SGA		LGA		5′ Apgar <7*		Neonatal death	
	N	Mean	Mean diff	N	OR	N	OR	N	OR	N	OR	N	OR	N	OR	N	OR	N	OR	N	OR	N	OR
		SD	95% CI	%	95%CI	%	95%CI	%	95%CI	%	95%CI	%	95%CI	%	95%CI	%	95%CI	%	95%CI	%	95%CI	%	95%CI
Spontaneous	7215	2426	Ref	575	Ref	3564	Ref	603	Ref	2859	Ref	4	Ref	193	Ref	683	Ref	703	Ref	226	Ref	142	Ref
		613		8.0		49.4		8.4		39.6		0.1		2.7		9.5		9.7		3.1		2.0	
Any assisted conception	1609	2302	**−58**	193	**1.35** #	907	1.12#	200	**1.32** #	699	1.10#	2	1.53	41	0.90	154	0.85#	117	0.88	41	0.77	25	0.78#
		639	**−109,−8**	12.0	**1.05,1.72**	56.4	0.96,1.30	12.4	**1.02,1.71**	43.4	0.93,1.30	0.1	0.15,15.9	2.6	0.64,1.27	9.6	0.69,1.04	7.3	0.70,1.12	2.6	0.51,1.16	1.6	0.45,1.35
Spontaneous conception, fertile	7016	2430	Ref	549	Ref	3457	Ref	578	Ref	2787	Ref	4	Ref	188	Ref	664	Ref	689	Ref	221	Ref	133	Ref
		609		7.8		49.3		8.2		39.7		0.1		2.7		9.5		9.8		3.2		1.9	
Spontaneous conception, IF DX***	166	2232	**−196**	24	**1.81** #	96	1.23#	23	1.62#	65	0.90#	0	1.85	5	0.99	17	0.93	11	0.57	5	0.88	9	**2.86**
		730	**−354,−37**	14.5	**1.0003,3.29**	57.8	0.83,1.81	13.9	0.86,3.05	39.2	0.58,1.41	0.0	0.17,20.1	3.0	0.41,2.37	10.2	0.51,1.72	6.6	0.22,1.44	3.0	0.31,2.53	5.4	**1.11,7.39**
Spontaneous conception, IF TX****	33	2574	−85	2	1.03	11	0.81	2	0.98	7	0.45	0	1.70	0	**0.64** ∧	2	0.87	3	0.43	0	0.76∧	0	1.69∧
		581	−304,134	6.1	0.17,6.04	33.3	0.34,1.95	6.1	0.17,5.62	21.2	0.14,1.43	0.0	0.16,18.5	0.0	**0.42,0.97**	6.1	0.28,2.72	9.1	0.02,8.28	0.0	0.42,1.38	0.0	0.76,3.74
Donor oocyte	16	2271	−104	2	1.42	10	1.41	4	2.90	8	1.18	0	1.54	0	0.35∧	1	0.35	1	0.72	0	1.32∧	†	-
		682	−575,368	12.5	0.16,12.9	62.5	0.32,6.20	25.0	0.55,15.4	50.0	0.27,5.14	0.0	0.12,19.0	0.0	0.05,2.45	6.3	0.04,2.78	6.3	0.14,3.71	0.0	0.31,5.67		
GIFT	212	2204	−125	32	1.69#	131	1.34#	31	1.45#	98	1.20	0	0.98	7	1.13	22	0.88#	9	**0.58**	9	1.34	7	1.65
		684	−270,20	15.1	0.95,2.99	61.8	0.93,1.92	14.6	0.77,2.74	46.2	0.81,1.78	0.0	0.09,10.8	3.3	0.54,2.39	10.4	0.56,1.37	4.3	**0.34,0.98**	4.3	0.56,3.20	3.3	0.50,5.49
IUI	138	2279	−75	22	**1.96**	84	1.36	21	1.74	53	0.88#	0	1.56	3	0.70	15	0.92	9	0.87	1	0.22	1	0.28∧
		644	−230,79	15.9	**1.06,3.62**	60.9	0.89,2.09	15.2	0.89,3.43	38.4	0.54,1.43	0.0	0.14,17.0	2.2	0.21,2.28	10.9	0.50,1.68	6.5	0.46,1.67	0.7	0.03,1.64	0.7	0.04,1.99
IVF with fresh-embryo cycles	543	2274	**−96** #	62	1.31#	316	1.23#	61	1.21#	252	1.22#	0	1.16	22	1.50	63	1.07	32	0.71	12	0.66	8	0.80#
		608	**−165,−27**	11.4	0.90,1.90	58.2	0.98,1.55	11.2	0.81,1.81	46.4	0.95,1.57	0.0	0.11,12.5	4.1	0.96,2.35	11.6	0.79,1.45	5.9	0.48,1.05	2.2	0.32,1.36	1.5	0.33,1.94
IVF with frozen-embryo cycles	147	2368	−40	16	1.35	68	0.76#	22	1.84	58	0.89#	0	1.00	1	0.25	9	0.54	12	0.75	4	0.85	0	0.77∧
		653	−189,109	10.9	0.64,2.86	46.3	0.50,1.17	15.0	0.95,3.55	39.5	0.55,1.44	0.0	0.09,11.1	0.7	0.03,2.06	6.1	0.26,1.12	8.2	0.38,1.50	2.7	0.25,2.95	0.0	0.21,2.80
ICSI with fresh-embryo cycles	380	2332	−42	43	1.30	211	1.05#	43	1.22	160	1.05	2	1.58	6	0.56#	28	**0.64** #	35	1.15	12	0.96	8	1.22
		653	−138,53	11.3	0.83,2.03	55.5	0.81,1.37	11.3	0.76,1.95	42.1	0.77,1.43	0.5	0.15,17.2	1.6	0.26,1.23	7.4	**0.43,0.94**	9.2	0.77,1.71	3.2	0.47,1.98	2.1	0.50,2.95
ICSI with frozen-embryo cycles	65	2523	122	6	0.77∧	28	0.68	8	1.00∧	26	1.00	0	1.40	0	0.49∧	4	0.57	12	**2.39**	1	0.43	1	0.78
		580	−64,307	9.2	0.24,2.45	43.1	0.39,1.18	12.3	0.37,2.70	40.0	0.50,2.02	0.0	0.13,15.7	0.0	0.19,1.27	6.2	0.23,1.41	18.5	**1.19,4.80**	1.5	0.05,3.73	1.5	0.08,7.59
Minimal medical intervention	45	2529	133	2	0.54	18	0.65	1	0.30	20	1.21	0	1.48	0	0.86∧	2	0.37	2	0.58	0	0.65∧	0‡	0.20∧
		491	−38,303	4.4	0.14,2.12	40.0	0.29,1.47	2.2	0.05,2.02	44.4	0.51,2.87	0.0	0.14,16.2	0.0	0.54,1.36	4.4	0.09,1.52	4.4	0.19,1.77	0.0	0.30,1.38	0.0	0.02,1.85
OI only	63	2213	−139	8	1.47	41	1.60	9	1.60	24	0.86	0	1.51	2	1.08	10	1.43	5	0.82	2	1.01		
		663	−324,46	12.7	0.54,4.03	65.1	0.85,3.03	14.3	0.61,4.16	38.1	0.41,1.78	0.0	0.14,16.6	3.2	0.27,4.32	15.9	0.64,3.23	7.9	0.19,3.55	3.2	0.25,4.11		

Odds ratios account for clustering within mother, and are adjusted for maternal age, parity, baby's sex and whether twins same-sex.

Bold print signifies p<0.05.

19 births were missing 5 minute Apgar score information.

^#^ Significant difference between effect for singletons and twins.

Model assumes common effect of twins on modality effects.

IF DX: Births to women who had a recorded diagnosis of infertility but no assisted conception treatment from a specialist clinic.

IF TX: Births as a result of spontaneous conception in women with a previous birth from assisted conception.

Data for neonatal death occurring in donor oocyte pregnancies collapsed in IVF and ICSI categories due to small numbers.

Data for neonatal death occurring in the groups minimal medical intervention and ovulation induction only combined due to small numbers.

Singletons and twins combined because of small numbers (comparator is twins).

Low birth weight (LBW): <2500 g; Very low birth weight (VLBW): <1500 g; Preterm birth (PTB): 32–37 weeks; Very preterm (VPTB): <32 weeks; Postterm: >41 weeks; SGA <10th percentile, Roberts [15]; VSGA <3rd percentile, Roberts [15]; LGA: >90th percentile, Roberts [15].

Compared to twin births in the spontaneous conception, fertile group, twins from GIFT were less likely to be LGA, twins from IUI were more likely to be VLBW, those from IVF with fresh-embryo cycles were born lighter, those born after ICSI with fresh-embryo cycles were less likely to be SGA and those born after ICSI with frozen-embryo cycles were more likely to be LGA.

## Discussion

In this large, population-based dataset, singleton births resulting from any assisted conception had almost double the risk of stillbirth of those conceived spontaneously. Liveborn infants from assisted conception were significantly more likely to be born preterm, be compromised on a range of indicators of birth size, and die in the neonatal period compared to their spontaneously conceived counterparts. These findings are consistent with those of recent studies [8], [16], with risk estimate magnitudes very similar to those in two earlier meta-analyses [4], [5]. However, our study represents a significant advance on previous studies as we were able to comprehensively demonstrate that the risks of adverse outcomes varied by type of assisted conception.

Among singletons, the increased risk of stillbirth was present for births resulting from fresh-embryo cycles of IVF or ICSI (but not frozen) and GIFT. Although an increased risk of stillbirth has been associated with IVF/ICSI relative to spontaneous conceptions and non-IVF treatments [17], our study is the first to demonstrate separate effects for the invasive assisted reproductive technologies, and to document an increased risk in women with a history of infertility but no treatment. We found very low birth weight, low birth weight, very preterm birth, preterm birth and neonatal death were markedly more common (ranging from a two to five-fold increase) and postterm birth less common among singletons conceived using IVF with fresh-embryo cycles than among those spontaneously conceived by fertile couples. Our results regarding birth size are consistent with previous studies demonstrating an association between IVF and smaller birth size [16], [18], [19]. However, the magnitude of the difference observed in this study (250 g reduction among IVF with fresh-embryo cycle births) is greater than in previous studies.

We also found increased risk of adverse outcomes among births from ICSI with fresh-embryo cycles, but the effects appeared considerably weaker than those seen for IVF. Studies of the Swedish IVF Registry [20] found no such differences between ICSI and IVF, even after taking cryopreservation effects into account, consistent with a clinical study [21]. However, the Swedish study pooled singletons and twins for the comparison of IVF and ICSI, which we suggest may attenuate observed effects. We also here confirmed the association between OI and an increased risk of low birth weight and preterm birth reported elsewhere [22]–[24].

Embryos transferred after cryopreservation may have better outcomes than those transferred fresh [25]–[28]. This apparent advantage has been attributed to the selection effect of freezing, whereby developmentally compromised embryos are less likely to survive, and to the placement of the cryopreserved embryo in a uterine milieu which has recovered from the effects of hormonal stimulation [25]. Although a previous systematic review [29] found no difference between frozen and fresh-embryo transfer, a recent meta-analysis found consistently better outcomes among pregnancies arising after frozen-embryo transfer [30]. However, neither took into account the use of ICSI. A Danish birth registry study found significant reductions in risk of preterm birth and low birth after cryopreservation that occurred in both IVF and ICSI, [26]. In the present dataset, we previously reported that cryopreservation was significantly protective against birth defects in ICSI, but not IVF, where the risks in fresh cycles were relatively low [13]. In the present study, cryopreservation reduced risk magnitudes for stillbirth, small birth size and early delivery outcomes for IVF, and eliminated those risks for ICSI. It is uncertain why cryopreservation may be more beneficial in ICSI than IVF treatment. However, this may reflect differences in the characteristics of patients accessing these treatments, as ICSI was generally used for male infertility and IVF for female infertility. Hence, IVF cycles may have a higher proportion of female-factor only patients where the selection effect of a freeze-thaw cycle may be diluted. Alternatively, there may be an ICSI-specific effect on growth patterns. We observed that frozen-embryo transfer was associated with increased likelihood of large size for gestational age among singletons born after ICSI. This association has been reported previously, albeit in studies that pooled IVF and ICSI cycles [31], [32]. However, caution is warranted in the interpretation of findings for the ICSI with frozen-embryo cycles group as the number of patients treated in this way is small, and data are pooled for singletons and twins for some outcomes. Further research is required to understand the cause of these possible risk differentials and their impact on longer term health outcomes, particularly with regard to the potential for perturbation of the fetal epigenome [33].

The contribution of maternal risk factors (other than maternal age) to the observed treatment effects is unclear, as we did not adjust for infertility aetiology. The apparent differences in risk between IVF and ICSI may be due in part to factors related to both specific infertility treatments and adverse pregnancy outcomes, such as obesity [34]. Although there is some evidence that the higher risk of stillbirth in assisted conceptions is explained by treatment type not cause of infertility [17], further research is needed to elucidate the influence of parental factors and treatment effects on the range of outcomes considered in this study.

We separated spontaneous conceptions to women diagnosed with infertility but never treated from those to women previously treated, and we consistently found that negative consequences were limited to those diagnosed but untreated. Some outcomes indicated serious disadvantage, such as a quadrupling in risk of stillbirth, a 7-fold increase in neonatal death and an over 250 g deficit in birth weight. Adverse effects of subfertility on birth size and gestational age at birth have been observed previously [7], [9], [35], [36]. We cannot exclude residual confounding by the use of poorly supervised clomiphene citrate, also associated with smaller birth size [22]–[24] as we have previously reported that of women with a history of infertility, approximately half use intensive therapies through specialist clinics, while others use clomiphene citrate as a first line treatment that is not recorded in the data available for this study [37]. This group of births should be studied further to identify their pattern of exposure to fertility treatments and maternal factors.

Twins conceived with any assisted conception treatment were slightly lighter than spontaneously conceived twins. They were modestly more likely to be born at very low birth weight, and to be born very preterm, and had the same risk of neonatal mortality. This supports existing observations of outcomes among twins conceived with infertility treatment [38]–[40]. There were few differences in outcomes among twins by type of assisted conception treatment.

This large population-based study linked all assisted conception births in the state to outcomes obtained from the birth register. Unlike many prior registry studies, our analytic approach accounted for clustering within woman (by sibship) and within twins [41].

Limitations include the absence of information about: changes of male partner during interpregnancy intervals; zygosity and chorionicity of twins; and whether twins and singletons were reductions of higher-order pregnancies. Data were not available for assisted conception pregnancies beyond 2002. While improvements in pregnancy outcomes after assisted conception have been noted in the intervening years, these are primarily attributable to reductions in multiple pregnancy [42], with a further potential contribution from increasing use of cryopreservation [32]. Nevertheless, births after assisted conception continue to have a range of adverse perinatal outcomes [43], and therefore the results of this study, particularly the stratified analyses, are likely to have continued relevance internationally. A further potential limitation is that specialists may prescribe OI medications outside of specialist IVF clinics in South Australia, so some OI pregnancies may have been misclassified as spontaneous, and risks associated with assisted conception attenuated. However, we have ascertained from a registry of prescription data that this could contribute less than 1% of all births and is therefore a minor source of error for outcomes in the general population, but may contribute to the excess risk for uncommon outcomes, such as a stillbirth, in the group described above as the infertile but untreated population.

### Conclusions

Use of any assisted conception was associated with poorer perinatal outcomes for singletons than their spontaneously conceived counterparts. Diagnosed untreated infertility was associated with poor perinatal outcomes, but treatment of infertility at a different time was not. Outcomes from ICSI with fresh-embryo transfer and from IVF with frozen-embryo transfer were better than those from IVF with fresh-embryo transfer. ICSI with frozen-embryo transfer, despite lower per-cycle take-home baby rates and per-transfer singleton live birth rates [44], [45] does not appear to have worse outcomes than spontaneous conception, suggesting the procedure may select for healthier embryos or reflect differences in the characteristics of patients who have access to cryopreservation. However this type of assisted conception was associated with a greater risk of macrosomia. OI alone modestly increased the risk of low birth weight and preterm birth, a concern given its longstanding tenure as a first-line therapy, and its frequency of use. The comprehensive series of disadvantages among births following infertility treatment warrants routine monitoring and aetiological research.

## Supporting Information

Table S1 **Categories of assisted conception treatments for the time periods 1986–1992, 1993–1999 and 2000–2002.**
(DOCX)
