# Supplementary material for: Perinatal Outcomes by Mode of Assisted Conception and Sub-Fertility in an Australian Data Linkage Cohort
Source: PLoS One. 2014 Jan 8;9(1):e80398. doi: 10.1371/journal.pone.0080398 (PMC3885393; doi:10.1371/journal.pone.0080398)
Supplement: Table S1 — Categories of assisted conception treatments for the time periods 1986–1992, 1993–1999 and 2000–2002. (DOCX) [file pone.0080398.s001.docx]

|  | 1986-1992  N (%)* | 1993-1999  N (%)* | 2000-2002  N (%)* |
| --- | --- | --- | --- |
| Donor oocyte | 20 (1.2) | 40 (1.4) | 25 (1.8) |
| GIFT | 350 (21.4) | 170 (5.9) | 16 (1.1) |
| IUI | 178 (10.9) | 430 (14.9) | 107 (7.5) |
| IVF with fresh-embryo cycles | 556 (34.0) | 636 (22.0) | 327 (23.0) |
| IVF with frozen-embryo cycles | 132 (8.1) | 330 (11.4) | 142 (10.0) |
| ICSI with fresh-embryo cycles | 0 (0) | 637 (22.0) | 452 (31.8) |
| ICSI with frozen-embryo cycles | 0 (0) | 140 (4.8) | 145 (10.2) |
| Minimal medical intervention | 263 (16.1) | 332 (11.5) | 115 (8.1) |
| OI only | 135 (8.3) | 177 (6.1) | 94 (6.6) |
| Total | 1634 | 2892 | 1423 |

*Figures are a proportion of the column totals
